# A Dynamic Modeling Approach to Estimate Nitrogen Loading in Coastal Bays on Cape Cod, Massachusetts, USA

**DOI:** 10.3390/w14101529

**Published:** 2022-05-10

**Authors:** Stephen Balogh, Kate Mulvaney, Nathaniel Merrill, Amy Piscopo

**Affiliations:** U.S. Environmental Protection Agency, Office of Research and Development, Atlantic Coastal Environmental Sciences Division, Narragansett, RI 02882, USA

**Keywords:** nitrogen loading, nitrogen abatement, equilibrium model, wastewater, groundwater, Cape Cod, estuaries

## Abstract

Solving estuarine water quality problems on Cape Cod, Massachusetts, or elsewhere, is difficult. Nitrogen from septic systems takes years to decades to travel by groundwater to estuaries, depending on local hydrogeology, meaning that nitrogen loading in future years may exceed current conditions. We created a dynamic nitrogen model of Cape Cod’s 54 estuaries to better understand 1. how past and present conditions, including legacy nitrogen in groundwater, influence future nitrogen loading, and 2. how different development and nitrogen abatement scenarios could have additional effects. We find that 43 of 54 estuaries are not in equilibrium with current watershed nitrogen loading levels; this increases to 52 of 54 under a buildout scenario. Watersheds contain up to 1000 tons of legacy nitrogen in groundwater; yet, we find that a rapid investment in source control successfully reduces nitrogen loading, revealing a wide range of potential outcomes that depend ultimately on the resources and attention invested in the problem.

## 1. Introduction

A majority of the small estuaries on Cape Cod (Barnstable County, Massachusetts, USA) have excess nitrogen (N) loading resulting in a suite of negative water quality impacts that include degradation of habitats, decreased water clarity, and more [1]. In these watersheds, most of the N entering the bay comes from human additions of waste into groundwater via septic systems [2–4]. Total maximum daily loads (TMDLs) have been set for more than 30 bays on Cape Cod to comply with the U.S. Clean Water Act and to improve their condition [5]. Usually, there are delays between N entering the groundwater and its ultimate delivery to the bay because of slow groundwater transport in the aquifer [1,6,7].

**Figure 1.**
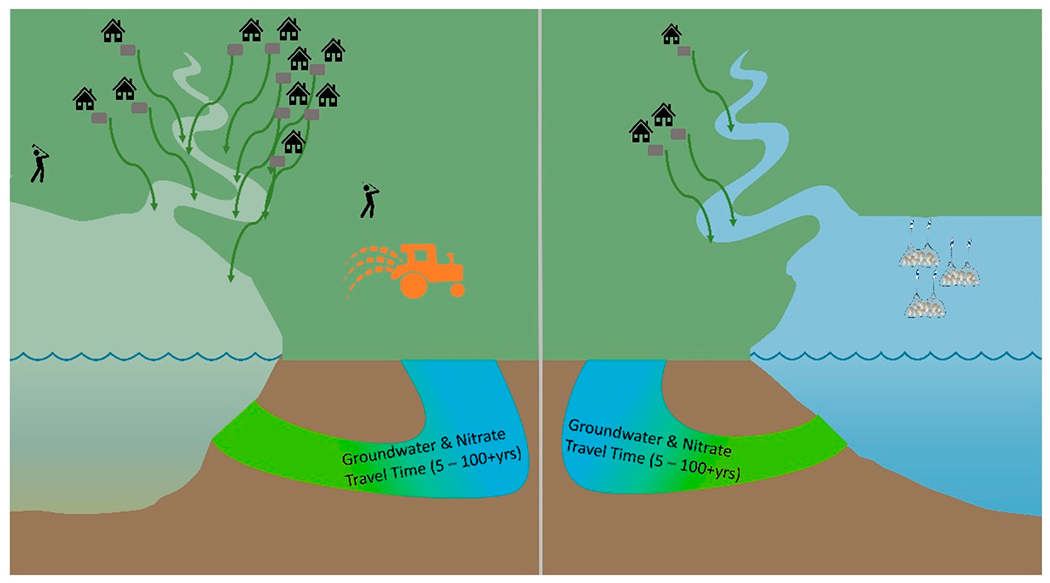
Nitrogen loading and processing in estuaries on Cape Cod, Massachusetts, USA. N loads to two example estuaries are primarily sourced from the household and commercial septic systems on Cape Cod. N loads flow via groundwater and surface waters to the bays. In the estuary on the left, water quality is more diminished as there is a higher N load from multiple sources (heavy use of septic systems, light agriculture, golf courses) in the watershed. The green color of the water indicates excess N loads and eutrophication. A less-developed watershed, such as the watershed on the right, has fewer sources of N loads (lower number of septic systems and less fertilizer use), and water quality is relatively better. Symbols from University of Maryland Center for Environmental Science Integration and Application Network (ian.umces.edu/media-library).

Nitrogen loading is often considered in an equilibrium state, meaning the annual N load to the groundwater from residential and commercial properties in the watershed is equal, or nearly equal, to the load entering the estuary each year, after considering natural attenuation (denitrification) in the ground and while passing through surface waters on its path to the estuary [8,9]. However, this may inaccurately represent loading in the estuary depending on the amount of N currently in groundwater, soil biogeochemistry, hydrology, geomorphology, and the social dynamics of the watershed. The amount of nutrients present in an estuary’s waters is dictated by multiple factors, including the nutrient load received from atmospheric deposition and from point and nonpoint sources within the estuary’s watershed [1,10] (Figure 1). The TMDLs for Cape Cod were designed to set targets for controllable load (also referred to as “watershed load”) which includes the sources that can be affected locally, such as wastewater or stormwater [5]. We focus our modeling on these controllable sources, while acknowledging that atmospheric deposition is changing over time as well, also affecting ecological conditions in the bays [11]. Similarly, we do not model changes in the N load from the bottom sediment in the bays themselves that affect in-water conditions [5].

Other important factors that influence nutrient concentrations in the bay include groundwater travel time, nutrient decay processes in the estuary and during transport to the estuary, and residence time [7]. Whether a home or business’s waste currently contributes to N loading is a function of its distance to nearby waterbodies, elevation, and year of construction. Groundwater travel times are much slower than overland water movements, and delays may result in a “legacy” supply of N traveling to the bay that has not yet affected water quality [12]. Likewise, there may be delays between interventions to improve N attenuation in the system and a reduction in N loading to the bay [6].

In most locations, N is primarily removed at the source through the use of traditional wastewater treatment plants and sewers (referred to in this paper as simply “sewers”) and, in some cases, advanced septic systems [13]; however, source-control measures may take decades to be installed and do not affect N that is already in the groundwater on its way to the bay. Additionally, using sewers may lead to inter-basin transfers of low N concentration but high-volume wastewater, as wastewater is removed from one watershed and piped to another watershed for treatment where it is discharged, albeit at a markedly lower concentration. The cost of meeting N reduction targets through additional sewers and wastewater treatment capacity has been estimated at USD 4–7 billion [14]. Alternatively, N can be addressed near or in the bay through intercepting or in situ techniques, for example, permeable reactive barriers or aquaculture (e.g., oyster and clam farming), but these technologies also have a number of social and technical limitations for use [13]. These limitations include uncertainty in cost and technical ability to reduce N, as well as the need for long-term monitoring and maintenance, regulatory challenges, and other social acceptance concerns.

Our research investigates the questions: (1) Given the long travel times, are estuaries on Cape Cod in equilibrium with groundwater N loading? (2) Which estuaries are not, and how many years until they reach equilibrium? (3) What is the difference between the N load at equilibrium and current loading? and (4) What is the effect of additional development on both the ultimate N load and time to reach equilibrium?

Using parcel-level building information and groundwater travel time models, we reconstructed historical N loads that had been added to the groundwater and model the contributions of groundwater N to the bays. This dynamic modeling approach to estimate N loading can help inform decision-makers about the current and future state of the watershed/bay system and provide insights about the application of a mix of source-control and in situ N removal technologies needed to improve bay water quality. The benefits to a regional, dynamic modeling approach are to anticipate changes in conditions over time and to determine which estuaries could be prioritized for funding or intervention type relative to one another.

## 2. Materials and Methods

There are 54 individual estuaries with associated watersheds and sub-watersheds on Cape Cod [15]. These 54 estuaries range in size from 0.25 to 4713 hectares, with groundwater contributing areas ranging in size from 30 to 8370 ha (0.3 to 83.7 km^2^). All the bays experience N loading from anthropogenic sources in the watershed, in addition to the atmospheric N deposited on land and directly on the water’s surface. These bays have long been important for productive littoral fisheries and nurseries for pelagic fish, and recreation and tourism are also valued by the Cape communities. There are approximately 200,000 year-round residents on the Cape, but the population swells to a peak of 650,000 in summer with tourists, second homeowners, and the seasonal workforce [15]. Millions of tourists visit the Cape each summer spending USD 1.1 billion in direct spending [16,17].

Specifically, to operationalize our N loading model (Figure 1), we used the following data:

1. Standardized (Level 3) parcel data maps for the 13 towns on Cape Cod from the Massachusetts Office of Geographic Information [18]. These maps provided the year that the property was built and its location.
2. Data from the Watershed Multi-variant Planner (MVP) tool [19], hereafter CCC-MVP. These data include whether the property is connected to sewer or has a septic system, within which watershed and sub-watershed the property is located, estimates of current N load and potential load after 20 years of development, and whether N flows from the site are subject to any natural attenuation when traveling to the bay. Total and attenuated N loads were calculated by the MVP effort by using parcel-level water use data and assumed effluent concentrations, as well as atmospheric deposition rates on the landscape and assumed landscape N attenuation rates.
3. U.S. Geological Survey (USGS) Plate: Ground-Water Recharge Areas and Travel Times to Pumped Wells, Ponds, Streams, and Coastal Water Bodies, Cape Cod, Massachusetts [20]. We used a digitized map of this plate produced by the Buzzards Bay National Estuary Program (Costa, personal communication). The USGS estimated groundwater travel times throughout Cape Cod and Costa digitized the map. USGS delineated coastal recharge areas using MODFLOW-2000 and SEAWAT models. The MODPATH particle tracking algorithm provided an estimate of groundwater travel time and delineated water table contributions to coastal and freshwater bodies [18].

Using GIS, we grouped and spatially joined these datasets to create a comprehensive geographic database of properties, their location, year built, N load, natural attenuation, and N load under a “built out” scenario of continued urbanization and development, which is incorporated into the upper-bound scenario described below [19].

Next, we used R programming language to simulate N loading over time. Using the year built and travel time associated with each property, we determined which properties would be contributing to N loading at the individual bays in every year. We subtracted any natural attenuation and summed the remaining N load to each embayment (see Appendix A for model details). We ran the model on an annual time step between year 1880 and 2150.

To understand the range of possible future states of N loading in Cape Cod bays and to estimate the legacy N in transit to the bays, we developed and simulated three scenarios: upper, lower, and legacy N. The first scenario used CCC-MVP buildout data [19] to incorporate additional potential sources of anthropogenic N from homes which may enter the groundwater. To explore the maximum possible N loading (upper-bound scenario), properties identified by CCC-MVP as having the potential for increased N load in the future are updated at random over a 20-year period from 2020 to 2040 (a 20-year time horizon was used by the Cape Cod Commission to formulate the buildout scenario [14]). The increased N load from these properties was only incorporated in future years where *year of update* + *travel time* < *current year.* This scenario did not include any additional source control of N. Currently, those properties not connected to sewer systems on the Cape are using on-site septic and cesspool systems that are not designed to mitigate N pollution. Despite the known embayment water quality issues, new construction is largely not required to mitigate the new contribution to the problem unless meeting size limits is deemed regionally important, which does not generally include single family homes [16,21].

In the lower-bound scenario, we modeled source control being implemented (rapidly) at all N-contributing, unsewered properties over a 20-year period from 2020 to 2040. To address uncertainty in which homes upgrade their septic systems, we used a Monte Carlo simulation with 1000 random iterations of the order in which properties are upgraded over this period. The N loads from properties with new source control were removed from annual contributions to the groundwater going forward; however, the N load from those properties (prior to the upgrade) that is in the groundwater which has not yet reached the bay was still included.

We then used our dynamic model to determine the amount of legacy N in transit to the bay, which we defined as all N that had entered the groundwater prior to 2020 that has not yet made it to a bay. To calculate this amount, we shut off all loading instantaneously in 2020 and tallied the annual N load reaching each bay from 2025 to 2150.

## 3. Results

**Figure 2.**
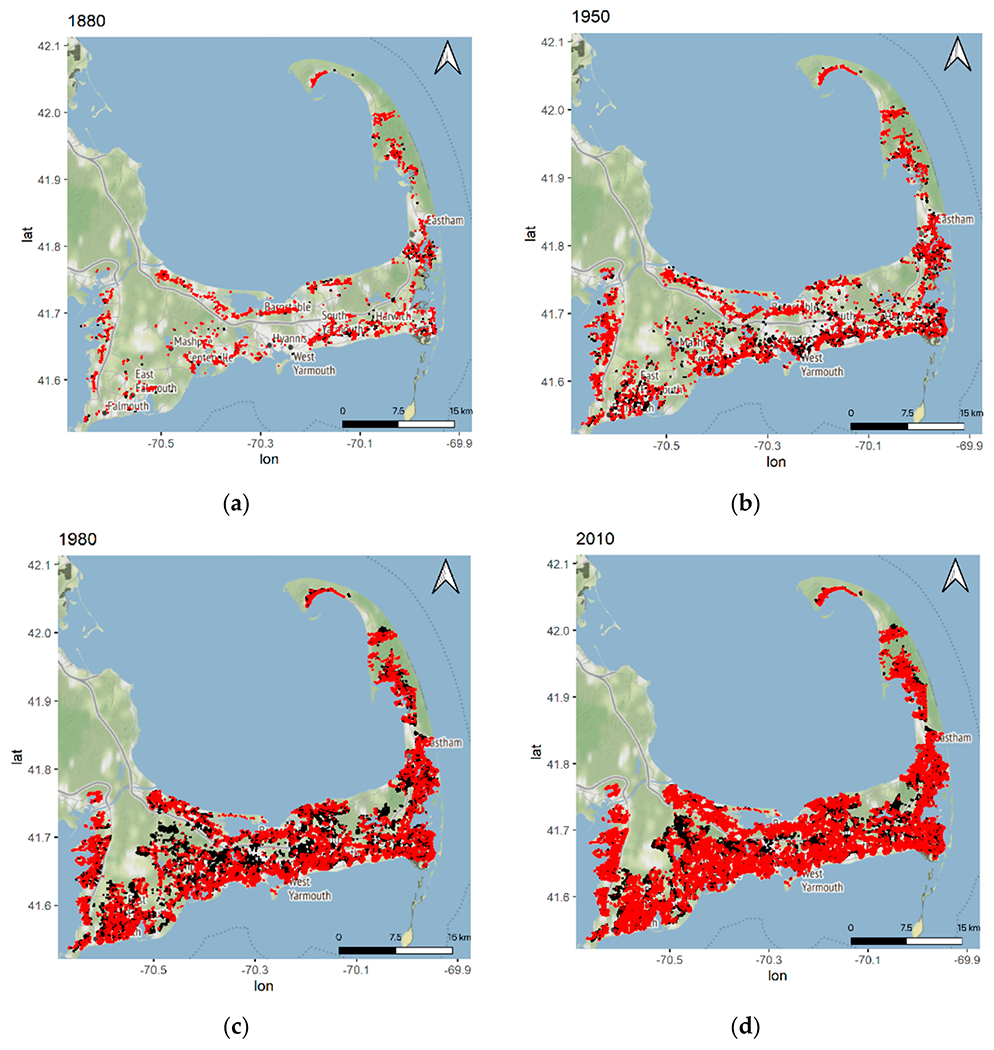
N load sources on Cape Cod from (**a**) 1880 to (**b**) 1950 to (**c**) 1980 to (**d**) 2010. Red dots indicate that N from that source is reaching the bay, while black dots indicate that the N from that source has not yet reached the bay by the year indicated in the map. Properties with direct discharge to the Atlantic Ocean are excluded.

**Figure 3.**
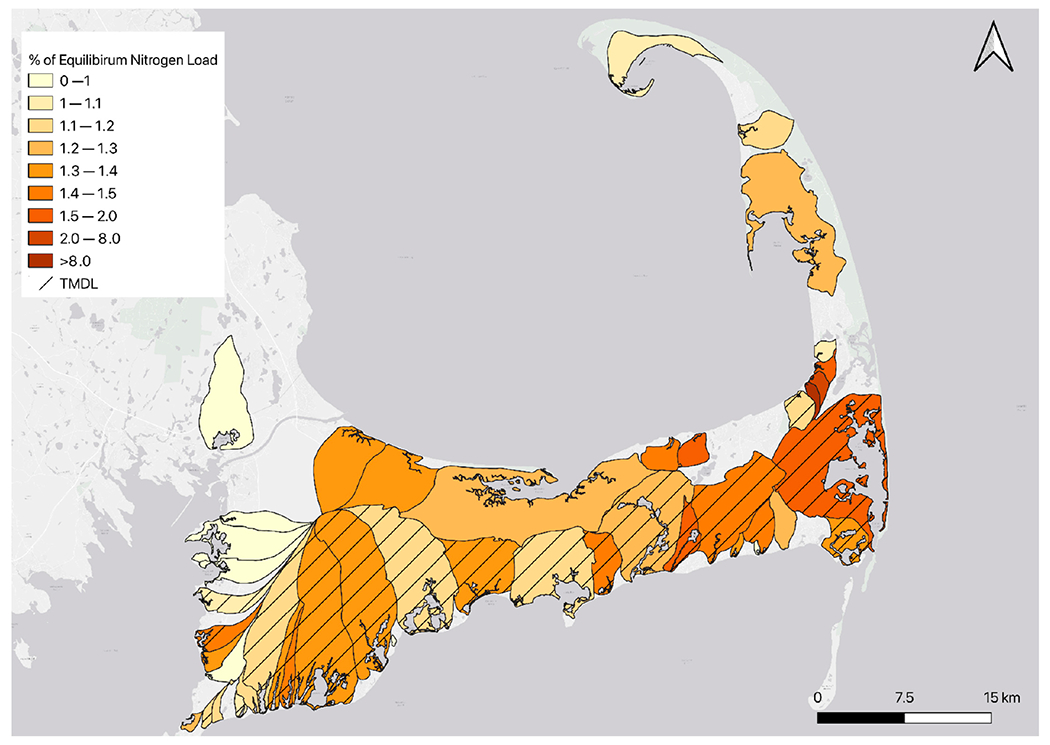
Map of the watersheds for each embayment. Watersheds in watershed loading equilibrium are indicated in pale yellow. Those not in equilibrium are shaded from light orange to dark brown based on their expected increase in N loading above current conditions (see legend). Watersheds with TMDLs are indicated with cross-hatching.

**Figure 4.**
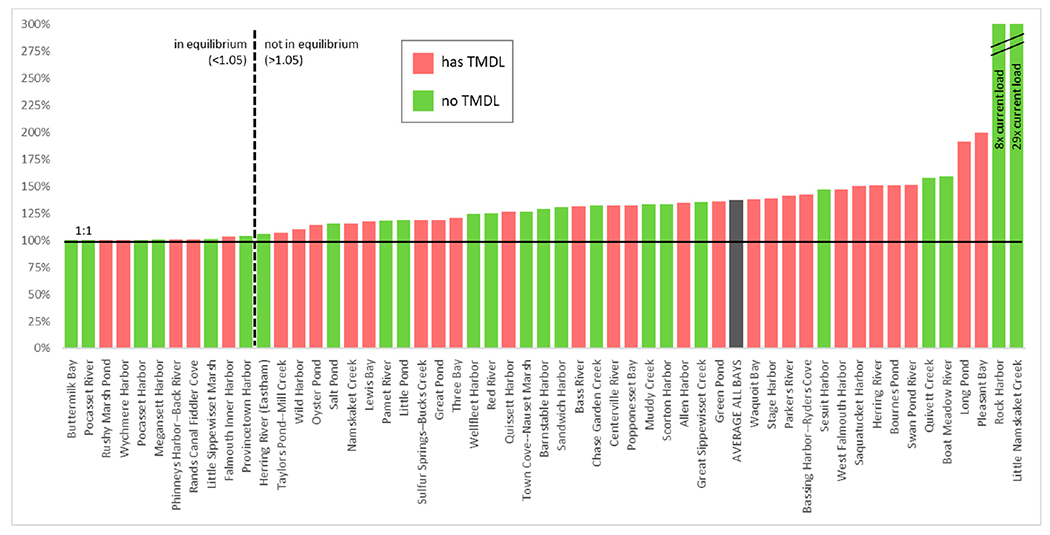
N loading in embayments on Cape Cod. Ratio of maximum future load (estimated) to current load. Bays at or near 1.0 are in equilibrium in terms of watershed load, while bays that exceed 1 are expected to experience greater N loading than current conditions. Rock Harbor (8× current load) and Little Namskatet Creek (29× current load) exceeded the scale of the *y*-axis of the chart.

**Figure 5.**
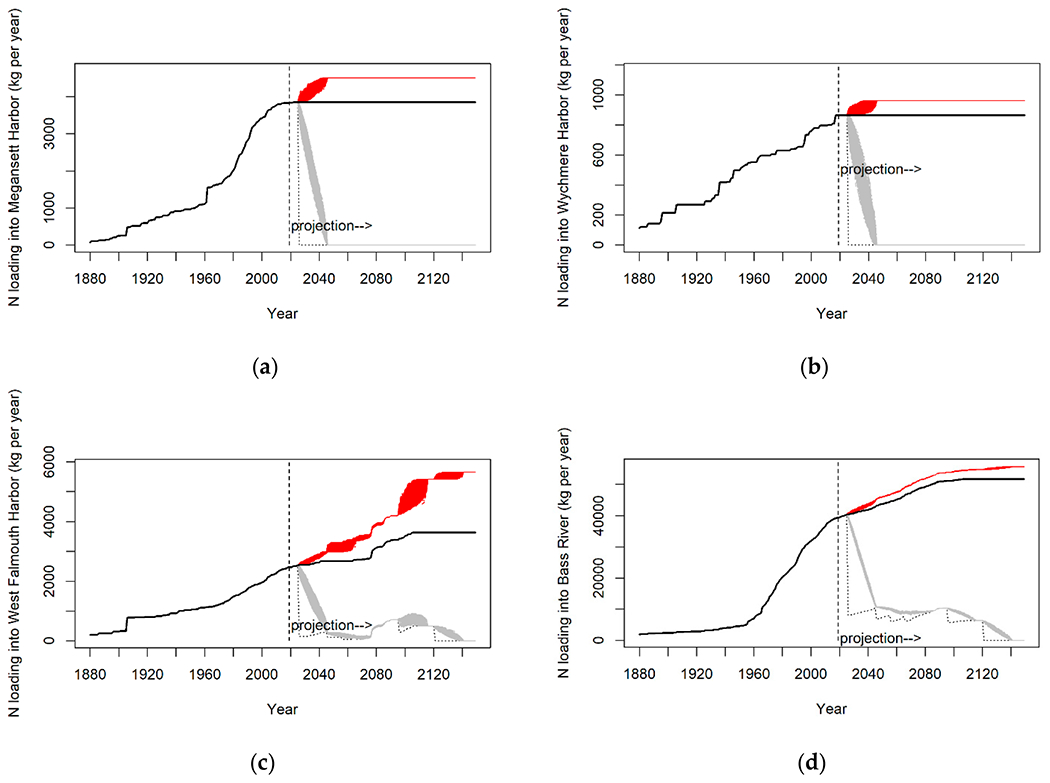
Example bays in equilibrium in terms of watershed N loading sources: (**a**) Megansett Harbor and (**b**) Wychmere Harbor N loading in embayments on Cape Cod, and example bays not in watershed loading equilibrium: (**c**) West Falmouth and (**d**) Bass River. N loading over time to select Cape Cod embayments. The vertical dashed line indicates the current year, to the right of the dashed line is the projected N load. The top two graphs depict bays in equilibrium where, all other things held equivalent, future loading ≈ current loading. The bottom two graphs depict bays where future N load will exceed current loading. For all four bays the results from the Monte Carlo analysis in the upper-bound scenario are indicated by red lines, and the lower-bound scenario results are indicated by gray lines. The thin dotted black line at the bottom is the legacy N scenario. Note: *y*-axis scale varies by estuary. See Appendix B for complete N loading time series results.

**Figure 6.**
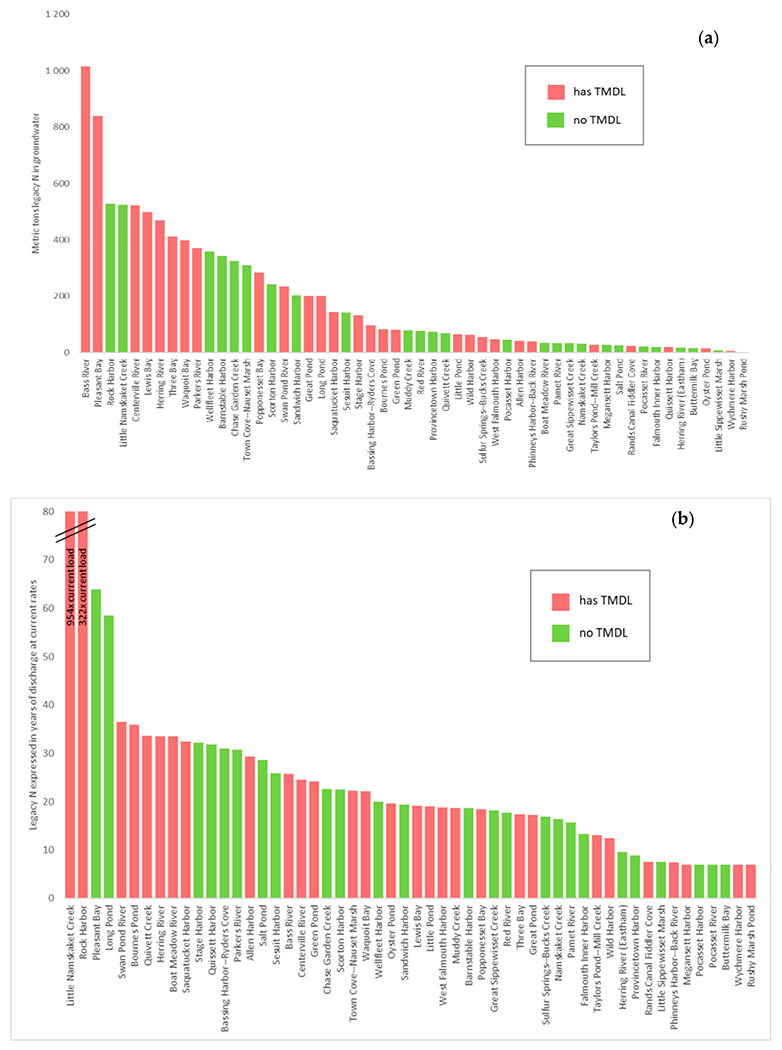
Legacy N load in groundwater in Cape Cod embayment watersheds (from 2025 to 2150). This was calculated by running a simulation where all N non-point sources were turned off in 2020 and the remainder of N in the system was allowed to travel over time to reach the respective bays. Expressed in (**a**) absolute units (tons N), (**b**) expressed in multiples of 2020 annual load. Bays with final TMDL in place indicated by red columns, and green columns indicate TMDL not required or not in place yet.

The number of sources of N has increased dramatically with nearly 33 times more properties existing today than in 1880 (Figure 2). The input of N from properties varies by several orders of magnitude in watersheds, and the average travel time from properties to their embayment varies from 5 to 47 years among the different watersheds (Table 1). Given the N loads and natural attenuation assumptions from the CCC-MVP dataset [15] and travel times from the USGS [20], we find that watershed N loading in 43 out of 54 watersheds is currently not in equilibrium with the actual load at the embayments, and the eventual annual N load at the estuary from current sources will be greater in the future than the load realized at present (Figures 3 and 4). Just over half (51% (22/43)) of these non-equilibrium watersheds have a TMDL in place (Figure 3). The average time to reach equilibrium, assuming no more buildout or abatement plans, is 73 years (min = 0, max = 99), and the average increase above current conditions for all bays is 1.36× current loading (min 0×, max 29× current load) (Figure 4). Examples of N loading plots for equilibrium and non-equilibrium bays are provided in Figure 5a–d (plots for all 54 watershed can be found in Appendix B). Nearly all (96% (52/54)) of the watersheds are expected to have increased loading under a development/buildout scenario, with the mean increase in loading per bay around 1500 kg of N per year (min 0.0, max 7500 kg). The mass of legacy N in transit to the bays ranges from 0.4 to 1015 metric tons of N, equivalent to between 7 to 954 years of N loading at current rates (Figure 6a,b).

## 4. Discussion

Our results indicate that there are a wide range of potential outcomes for N loading from non-point sources across Cape Cod estuaries. Even in the best-case scenario, the region is dealing with a multi-generational problem. Which of these futures will be realized will depend on when, where, and to what degree people intervene in the system [6]. We find that under business-as-usual conditions, the watershed loading to most embayments may get worse before it gets better. If development continues as expected, maximum N loading will be even greater, take longer to reach equilibrium conditions in those watersheds, and may require additional N removal interventions in the watersheds or bays.

The effects of adding source control, either wastewater collection and treatment capacity or advanced septic systems, reduces N loading into the groundwater, but groundwater transport delays and legacy N will remain a factor in many estuaries (24 out of 52 embayments have more than 100 tons of legacy N present in groundwater). Additionally, there may be additional delays as there may be a latency period between achievement of lower N concentrations and estuarine recovery. The Chesapeake Bay system demonstrates that perceptible improvements in water quality (oxygen, water clarity, chlorophyll a, and submerged aquatic vegetation) may be achieved quickly after reductions in nutrient and sediment loads [22], while other attributes of the bay ecosystem, e.g., return of charismatic species of birds, fish, and crabs, are expected to take years to meet outcome goals. Adding traditional wastewater capacity may also create new challenges for towns as the high-quantity, low-N-concentration effluent needs to be discharged after treatment, resulting in a new loading source or increased loading for an existing wastewater treatment plant. While many of the towns have been investigating wastewater treatment plants’ siting and increases, guidance is still being developed to integrate thinking across watersheds (for example, [23]).

Other sources of N exist besides the watershed loading from wastewater treated only by septic systems that affect the water column of the estuaries, including atmospheric deposition. As a source of N, atmospheric deposition has been lessening due to air quality improvements, which have potentially offset increases in groundwater N loading on Cape Cod over the last 30 years [11]. Similarly, the sediments on the bottom of the estuaries are sources of legacy N that affect the water column [9], while also creating additional lags between watershed loading improvements and results in the water. We also do not include any climate change effects on the system but acknowledge they will likely impact multiple processes. The effects of climate change on the groundwater transport of N, attenuation on the landscape, atmospheric deposition and sediment remobilization, and N assimilation in the estuary are important future research topics with implications for how pollution control efforts can be expected to ultimately affect in-water conditions.

Taking a Cape-wide, dynamic modeling approach to estimate N loading demonstrates the notion that there is not a “one size fits all” approach appropriate for all towns and all embayments to manage N pollution. As shown in Table 1, there are considerable ranges across the estuaries in terms of when properties were developed, mean groundwater travel times, and annual N load, which are mitigated or are accentuated by attributes of the embayment itself. Embayments that are at or near watershed loading equilibrium and have relatively short average groundwater travel times in the watersheds might be best served by a focus on source control, especially for those properties closest to the bay waters. Other watersheds and embayments that are further from equilibrium, may also incorporate in situ or intercepting technologies alongside more traditional source control measures to meet loading goals. Examining the effects of source control using a dynamic model highlights the benefits of also considering in situ (e.g., aquaculture, wetlands restoration) and intercepting (e.g., permeable reactive barrier) interventions as part of a comprehensive water quality management plan. These alternative approaches may partly address the delays in source control implementation and effect, as well as the legacy nutrients [6].

To meet loading reduction requirements for an estuary, N is considered from a “net removal” approach, meaning that removing a kilogram of N at the source does not always equate to a reduction of one kilogram from entering the bay, especially if that property’s groundwater passes through wetlands, ponds, or other natural sources of denitrification along the way. Similarly, when considering the cost or efficiency of additional sewer capacity, at first glance, tertiary treatment to remove nutrients from influent seems very effective, reducing it from 50 mg/L (average influent concentration) to as low as 3 mg/L [24] (pp. 5–118). However, if the N entering the groundwater from a property with a Title V septic system is reduced to 35 mg/L after traveling through the soil just beyond the leach field, and the groundwater passes through a pond and a wetland on its way to the bay, then the realized N concentration entering the bay from that property might be 12.5 mg/L. Therefore, the net reduction achieved by adding that property to the sewer might only be 9.5 mg/L (12.5–3), rather than the more impressive-sounding 47 mg/L reduction from untreated wastewater (50 mg/L) to tertiary-treated effluent (3 mg/L). The same caveat applies to estimating abatement benefits of innovative/advanced (I/A) septic systems, where the baseline loadings already include denitrification after the septic system in addition to watershed attenuation en route to the bay.

Complicating an already multidimensional planning problem, some watersheds and estuaries are split between neighboring towns, leading to the need for regional public processes to encourage sharing the pollution problem and cleanup. This potential cooperation between towns may lead to shared wastewater infrastructure and monitoring programs and result in efficiencies of scale, but also may lead to delays in implementing plans as agreements are settled [16,25]. The legacy N pollution may also obscure the easy attribution of responsibility by adding a time dimension.

We combined existing models of the system, loading from the MVP dataset and groundwater travel times from the USGS. These each carry their own uncertainties, and different entities on Cape Cod use variations of these parcel loading and travel time estimates for different applications [24]. Nutrient attenuation by wetlands and ponds is a particular area of uncertainty that, if clarified, would improve our understanding of these systems. We acknowledge the potential for refinements of these regional datasets with potential for updated loading and travel time estimates.

We simulated nutrient abatement plans using a randomization of properties being upgraded with advanced septic systems or connection to new or existing wastewater treatment via sewers. These programs will likely come in the form of spatially and temporally contiguous sets of houses and neighborhoods. Incorporating real examples from town sewer plans across the Cape is left for future work. Similar uncertainty exists about the timing and extent of homes added in a buildout scenario. Actual growth in housing from 2010 to 2018 was 2.5% and ranged from 0% to 0.5% on a year-over-year basis [26], equivalent to a 0.31% compound annual growth rate (CAGR). The buildout assumptions in the CCC-MVP spreadsheet [14] included additional N loading from 12.1% of properties over the 20-year buildout period, a 0.53% CAGR. Therefore, our buildout scenario may overestimate the rate and ultimate number of homes added in the next 20 years on the Cape somewhat, but this still allows for the exploration of the upper limits of N loading.

## 5. Conclusions

The temporal dynamics of the system, highlighted in this work, do not change the fundamental problem at hand: there is a need to reduce sources of N in the estuary watersheds. What the time dimension contributes is a nuanced understanding of the potential trajectories of loading and resultant environmental conditions. However, the paths that the watersheds and estuary systems do ultimately take is in the control of the towns, depending on the extent and timing of sewering and septic upgrades the town chooses to follow, combined with the blend and location of any alternative approaches.

It took decades and generations of development and habitation to create the current pollution problem and it will take decades to reverse the impacts. As such, there will be ample opportunities and need to reassess science and management approaches; however, in the meantime, pollution continues to be added to the groundwater traveling to the estuaries.

## Supplementary Material

Supplement1

Supplement2

## Figures and Tables

**Table 1.** Quantity and age of properties in embayments, and total estimated annual watershed nitrogen load to estuaries in equilibrium.

	Embayment	Property Count	Mean Year Built	Std. Dev. Year Built	Mean Travel Time (Years)	Std. Dev. Travel Time (Years)	Annual Maximal N Load (kg)
1	Allen Harbor	291	1959	40.6	26.4	32.1	2399
2	Barnstable Harbor	3626	1963	50.7	19.6	28.2	27,754
3	Bass River	9532	1965	32.8	24.5	30.7	61,673
4	Bassing Harbor–Ryders Cove	1102	1971	27.7	29.5	33.8	5501
5	Boat Meadow River	324	1975	36.5	31.6	33.6	1887
6	Bournes Pond	1041	1974	25.6	32.6	34.8	4226
7	Buttermilk Bay	371	1959	33.7	5.0	-	2226
8	Centerville River	6738	1969	28.4	25.2	30.5	34,161
9	Chase Garden Creek	3068	1970	35.7	25.2	33.4	22,586
10	Falmouth Inner Harbor	399	1952	28.9	13.5	12.5	2097
11	Great Pond	4233	1972	24.2	17.8	27.1	15,863
12	Great Sippewisset Creek	475	1973	37.8	16.5	20.3	2881
13	Green Pond	1316	1973	21.5	29.2	34.7	5492
14	Herring River	3999	1967	40.4	30.5	34.8	25,525
15	Herring River (Eastham)	354	1979	22.9	9.2	12.6	2068
16	Lewis Bay	6131	1964	25.4	19.9	25.5	36,369
17	Little Namskaket Creek	250	1960	49.4	47.4	38.5	15,905
18	Little Pond	1180	1964	19.0	14.1	20.2	4980
19	Little Sippewisset Marsh	262	1976	21.5	8.4	5.3	1309
20	Long Pond	1254	1970	9.5	42.8	39.8	7768
21	Megansett Harbor	722	1956	39.5	5.0	-	3845
22	Muddy Creek	1157	1976	30.5	18.4	25.4	7028
23	Namskaket Creek	590	1978	29.2	16.6	16.0	2966
24	Oyster Pond	153	1964	28.1	21.9	25.9	1010
25	Pamet River	546	1948	63.3	17.4	23.1	2960
26	Parkers River	3134	1968	14.2	30.3	34.9	19,952
27	Phinneys Harbor–Back River	1162	1960	40.6	6.1	4.1	5505
28	Pleasant Bay	4661	1966	42.3	32.6	35.4	30,781
29	Pocasset Harbor	1571	1954	38.7	5.4	1.9	6982
30	Pocasset River	709	1961	34.5	5.1	1.1	3159
31	Popponesset Bay	6496	1983	21.5	22.6	30.3	24,398
32	Provincetown Harbor	1405	1935	60.8	7.2	10.3	9191
33	Quissett Harbor	140	1938	52.3	41.7	38.5	1012
34	Quivett Creek	554	1969	46.3	32.1	34.0	3517
35	Rands Canal Fiddler Cove	498	1965	44.8	7.0	5.8	3429
36	Red River	1266	1971	26.0	18.3	26.4	6341
37	Rock Harbor	342	1941	58.7	23.8	27.3	13,772
38	Rushy Marsh Pond	6	1973	27.2	6.7	4.1	63
39	Salt Pond	235	1953	41.4	20.0	18.3	1389
40	Sandwich Harbor	1871	1954	57.9	19.8	29.2	14,575
41	Saquatucket Harbor	1086	1962	45.6	36.2	34.6	8045
42	Scorton Harbor	2287	1978	28.6	25.0	28.9	15,249
43	Sesuit Harbor	1376	1972	36.0	27.9	33.2	9363
44	Stage Harbor	1389	1959	45.1	33.8	37.0	6796
45	Sulfur Springs–Bucks Creek	1030	1970	30.7	17.4	24.3	4640
46	Swan Pond River	2052	1964	24.4	31.3	35.7	11,721
47	Taylors Pond–Mill Creek	685	1961	44.0	11.7	11.8	2913
48	Three Bay	6795	1975	32.5	22.3	30.1	33,954
49	Town Cove–Nauset Marsh	2806	1963	43.1	26.4	31.8	19,803
50	Waquoit Bay	5897	1980	23.0	23.0	31.8	28,779
51	Wellfleet Harbor	3882	1964	47.3	21.2	27.1	25,333
52	West Falmouth Harbor	705	1960	49.8	22.8	33.6	3945
53	Wild Harbor	1271	1976	29.0	13.3	15.4	7021
54	Wychmere Harbor	129	1916	64.1	7.3	4.2	1042

## Data Availability

The data used in this paper are openly available at https://doi.org/10.5281/zenodo.5180583 (accessed on 3 May 2022).

## Appendix A

The modeling approach taken in this paper follows the methodology in Merrill et al. [6]. The equations associated with the approach have been modified below to reflect the specific N abatement effort under consideration in the paper (source control) and also the specific scenarios considered in this paper: buildout, rapid intervention scenario, legacy N.

For any given embayment, the load, *L*(*y*), entering that embayment at year, *y*, is the sum of *n* individual loading sources, *i* (Equation (A1)).

(A1) $$L\left(y\right)=\sum_{i}^{n}N_{i}\;Sk_{i}.$$

The terms in this equation are defined as follows:
*N_i_*—is the N load from source *i*. *S*—is the impact of the source control intervention of source *i* on the load *N_i_*. *k_i_*—is the natural attenuation of source *i*.

There is a time delay from source *i* to estuary of *τ_i_* (i.e., travel time). In other words, each source *i* has an associated load *N_i_*, and the travel time *τ_i_* of the load from the source to the embayment determines whether the load is presently (i.e., at any present year *y*) contributing to the load in the embayment. If the present year *y* is less than the year the source began producing load, *yo_i_*, plus the travel time *τ_i_* of a source *i*, then that source load has not yet reached the embayment, meaning that (A1) is subject to:

(A2) $$N_{i}=\{\begin{array}{c} N_{i}\;\text{for}\;y>\tau_{i}+yo_{i}\\ \text{0 otherwise} \end{array}.$$

where:
*yo_i_*—is the year at which the source began producing load. *τ_i_*—is the travel time of the load from source to estuary.

For instance, if the travel time *τ_i_* from a source *i* (i.e., a home with a septic system) to the embayment is 25 years (*τ_i_* = 25 years), then the load from the home is contributing to the load in the estuary at all years greater than or equal to *y* = *yo_i_* + 25 years.

Furthermore, the impact of a source control intervention *S* occurring at time *yu_i_* does not take effect at the estuary until year *y* = *yu_i_* + *τ_i_* because the N in the ground before source control occurred is still transported by groundwater to the embayment at all years between *yu_i_* and *yu_i_* + *τ_i_*. This means that (A1) is also subject to

(A3) $$S=\{\begin{array}{c} 1\;\text{for}\;y<\tau_{i}+yu_{i}\\ \text{0 otherwise} \end{array}.$$

where:

*yu_i_*—is the year that a source control upgrade takes place at source *i*.

We assume the source control intervention to remove 100% of the N at the source, as reflected by the 0 in Equation (A3).

The three scenarios investigated in this paper are represented as modifications to the boundary conditions of Equation (A1), implemented as follows. For the buildout scenario, the N load *N_i_* of a subset, the *n* N sources are increased to reflect new loads in the watershed. The times at which the subset of source loads are increased are decided by a uniform random distribution between the years of 2020 to 2040. Source control is not considered in this scenario; thus, the *S* term in Equation (A1) does not impact the load term (always equal to 1).

For the rapid intervention scenario, source control is implemented at all unsewered properties. The *yu_i_* variable in Equation (A1) represents when source control occurs; for the *n* total sources, *yu_i_* is chosen at random (uniformly) between the years of 2020 and 2040.

For the legacy N scenario, all sources are “turned off” to calculate the legacy N in the groundwater. This is implemented in Equation (A3) by setting *yu_i_* to 2020 for all sources. Loading still occurs at the embayment until *y* = *yu_i_* + *τ_i_* for all sources. We tallied the sum of all sources where *y* < *yu_i_* + *τ_i_* for each year, and then summed the loading for all years to estimate the total legacy N in groundwater.

## Appendix B

Plots for N loading under all scenarios for 54 estuaries in Cape Cod, 1880 to 2150. The vertical dashed line indicates the current year; to the right of the dashed line is the projected N load. For all bays, the results from the Monte Carlo analysis in the upper-bound scenario are indicated by red lines, and the lower-bound scenario results are indicated by gray lines. The thin dotted black line at the bottom is the legacy N scenario. The 54 estuaries are included in alphabetical order, with each estuary denoted in the *y*-axis label. Note: *y*-axis scale varies by estuary.
